# Lactate dehydrogenase is an indicator for outcomes of short-term and long-term in septic patients

**DOI:** 10.1371/journal.pone.0337213

**Published:** 2025-12-08

**Authors:** Zhao Zeng, Cuirong Guo, Fengning Tang, Ning Ding

**Affiliations:** Department of Emergency Medicine, The Affiliated Changsha Central Hospital, Hengyang Medical School, University of South China, Changsha, China; Pescara General Hospital, ITALY

## Abstract

**Objective:**

The association between lactate dehydrogenase (LDH) and clinical outcomes in sepsis was explored based on MIMIC-IV database.

**Methods:**

This was a retrospective study. Models including unadjusted model and adjusted models were performed for exploring the association of LDH with 30-day mortality and 1-year mortality. The smooth fitting curves were constructed by using generalized additive model. The predictive value of LDH for clinical outcomes in sepsis was evaluated. The statistical software of EmpowerStats (http://www. empowerstats. com) and R (http://www.R-project.org) were applied for analysis.

**Results:**

6775 sepsis patients were included. After adjusted for all potential confounders, for every 100 IU/L increment in LDH, the risk of 30-day mortality and 1-year mortality increased by 11% (odds ratio (OR)=1.11, 95%CI:1.08–1.13, P < 0.0001) and 12% (OR=1.12, 95%CI:1.09–1.14, P < 0.0001), respectively. The areas under the ROC curve of LDH for predicting mortalities of 30-day and 1-year were 0.667 (95%CI:0.652–0.681) and 0.646 (95%CI: 0.632–0.660), respectively.

**Conclusion:**

LDH was positively correlated with 30-day and 1-year mortalities in sepsis and the relationship was nonlinear.

## Introduction

Sepsis, as a common disease with poor outcomes and substantial economic burden, has been a worldwide health problem for several decades [1]. In recent years, more than 30 millions sepsis cases per year have been diagnosed globally with near 20% mortality [2]. Even in sepsis survivors, one in five would be re-admitted in hospital within one month after discharging [3]. One recent study in Spain including 311,674 records of 288,211 sepsis patients concluded that the death rates of sepsis and septic shock were 23.3% and 47.9%, respectively [4]. Moreover, the mortality of sepsis patients admitted in intensive care units (ICUs) was significant higher [5,6]. A national cross-sectional survey on ICUs in China found that a 90-day mortality of septic shock was over 50% [7]. For those sepsis patients with acute kidney injury, the outcomes were even worse [8].

LDH is an important enzyme for glycolysis and plays a significant role in cell metabolism [9]. Researches have revealed that in lactate metabolic cycle, LDH enables to promote the reversible conversion of pyruvic acid to lactate acid and serves as a biomarker of acute injury and increased membrane permeability in cells [10,11]. Clinical and experimental studies also showed that elevated serum level of LDH was associated with platelet activation, hypoxia, and angiogenesis [12–14], which may explain the close relationship between LDH and different diseases.

LDH has been applied as a prognostic indicator in multiple diseases including stroke [15], aortic dissection [16], infection [17]and cancer [18]. One large-scale population research based on the clinical laboratory of the Affiliated Hospital of Qingdao University investigated 172,933 patients with over 40 different diseases compared with 9528 healthy participants and revealed that LDH was a biomarker for common diseases [19]. Moreover, LDH, as an biomarker of inflammation, was also linked with complications and outcomes in COVID-19 [20]. In neonatal sepsis patients, LDH was identified as a risk factor for 28-day mortality [21]. One recent study including two large database showed that higher levels of serum LDH were significantly associated with higher risk of in-hospital mortality in sepsis [22].

LDH is capable of being a good predictor due to its easily accessibility and applicability in clinical practice. In this study, we aimed to investigate the relationship between LDH with clinical prognosis in sepsis based on a large public database.

## Methods

### Database and definitions

This study was performed based on the Medical Information Mart for Intensive Care IV(MIMIC-IV) (https://mimic.mit.edu/iv/) database. MIMIC-IV records all clinical and laboratory data of all the patients admitted in the ICUs of the Beth Israel Deaconess Medical Center (2008–2019) [23,24]. Sepsis patients in MIMIC-IV were included in the present study.

Sepsis was confirmed on the basis of the Sepsis 3.0 definition which the inclusion criteria were the presence of infection and a sequential organ failure assessment (SOFA) score ≥ 2 [25]. The International Classification of Diseases (ICD) 9th and 10th editions (ICD-9 and ICD-10 codes: ‘99592’, ‘A419’, ‘A4159’, ‘A4150’, ‘R6520’, and ‘R6521’) were utilized for the diagnosis of sepsis. According to the aim of the study, exclusion criteria were as follow: 1) missing data of LDH; 2) patients with missing data >5% variables; 3) less than 18-year-old.

### Ethics approval and consent to participate

This study was conducted in accordance with Good Clinical Practice (Declaration of Helsinki 2002). MIMIC-IV was an anonymized public database. To apply for access to the database, we passed the Protecting Human Research Participants exam (No.32900964). The project was approved by the institutional review boards of the Massachusetts Institute of Technology (MIT) and Beth Israel Deaconess Medical Center (BIDMC) and was given a waiver of informed consent.

### Data extraction and variables

PostgreSQL 9.6 software was utilized for data extraction from MIMIC-IV. General variables including age, gender, length of stay (LOS) in ICU and hospital, 30-day mortality and 1-year mortality were extracted. Variables in the first 24 hours after admission including comorbidities(renal disease, hypertension, diabetes, coronary artery disease(CAD)), systolic blood pressure (SBP), heart rate (HR), respiratory rate (RR), diastolic blood pressure (DBP), anion gap(AG), aspartate aminotransferase (AST), total bilirubin, alanine aminotransferase(ALT), total calcium, hematocrit, LDH, prothrombin time (PT), creatinine, thrombin time (TT), hemoglobin, lactate, urea nitrogen, red blood cells(RBC), platelet (PLT), white blood cells (WBC) and sodium were also extracted and analyzed. The scores of chronic health evaluation (APACHEII) and SOFA of each patient were extracted. If one variable had several records in 24 hours after admission, only the first record was enrolled.

### Statistical analysis

All the patients with sepsis were distributed into different four groups based on quartiles of LDH (25% quartile, 50% quartile and 75% quartile; Q1: < 197IU/L, Q2:198–268 IU/L, Q3:269–392 IU/L, Q4 > 393IU/L) (Table 1). General characteristics of the cohort were expressed as medians (continuous variables) and percentages or frequencies (categories variables). The comparison of different variables between LDH quartiles groups was implemented by Mann–Whitney U-test or Chi-squared test. Samples with missing data more than 5% for individual variable are excluded. For those missing data no more than 5%, multiple imputation was used to estimate missing values for each variable.

**Table 1:** Different variables between groups based on LDH(quartiles).

LDH(IU/L)(quartiles)						
Variables	Total	Q1(< 197)	Q2(198–268)	Q3(269–392)	Q4(>393)	P-value
Number	6775	1685	1697	1699	1694	
Age(years)	66.00 (55.00-77.00)	64.00 (54.00-75.00)	68.00 (56.00-79.00)	67.00 (56.00-78.00)	65.00 (54.00-75.00)	<0.001
**Gender(n,%)**						0.254
Male	3758 (55.47%)	965 (57.27%)	945 (55.69%)	935 (55.03%)	913 (53.90%)	
Female	3017 (44.53%)	720 (42.73%)	752 (44.31%)	764 (44.97%)	781 (46.10%)	
**Comorbidities(n,%)**						
Renal disease	303 (4.47%)	63 (3.74%)	73 (4.30%)	100 (5.89%)	67 (3.96%)	0.010
CAD	600 (8.86%)	129 (7.66%)	151 (8.90%)	157 (9.24%)	163 (9.62%)	0.207
Diabetes	190 (2.80%)	51 (3.03%)	44 (2.59%)	54 (3.18%)	41 (2.42%)	0.499
Hypertension	1370 (20.22%)	350 (20.77%)	355 (20.92%)	312 (18.36%)	353 (20.84%)	0.182
**Variables**						
HR(beats/min)	97.00 (83.00-112.00)	95.00 (82.00-111.00)	97.00 (82.00-112.00)	96.00 (83.00-111.00)	99.00 (84.00-114.00)	0.002
SBP(mmHg)	110.00 (97.00-128.00)	109.00 (96.00-125.00)	111.00 (98.00-128.00)	112.00 (98.00-131.00)	111.00 (98.00-127.00)	0.003
DBP(mmHg)	63.00 (53.00-74.00)	62.00 (52.00-72.00)	63.00 (53.25-73.00)	62.00 (52.00-75.00)	64.00 (53.00-76.00)	0.011
RR(beats/min)	21.00 (17.00-25.00)	20.00 (17.00-24.00)	20.00 (17.00-25.00)	21.00 (17.00-25.00)	22.00 (18.00-26.00)	<0.001
LDH(IU/L)	268.00 (197.00-391.50)	165.00 (144.00-181.00)	229.00 (212.00-247.00)	312.00 (287.00-345.00)	570.50 (459.00-854.75)	<0.001
AG(mmol/l)	16.00 (13.00-19.00)	15.00 (12.00-17.00)	15.00 (13.00-18.00)	16.00 (14.00-19.00)	17.00 (14.00-21.00)	<0.001
Total bilirubin(mg/dL)	0.70 (0.40-1.80)	0.60 (0.30-1.30)	0.70 (0.40-1.50)	0.80 (0.40-1.90)	1.00 (0.50-2.40)	<0.001
Total calcium(mg/dL)	8.10 (7.50-8.60)	8.10 (7.60-8.70)	8.10 (7.60-8.60)	8.10 (7.50-8.70)	8.00 (7.40-8.60)	<0.001
Creatinine(mg/dL)	1.30 (0.90-2.20)	1.20 (0.80-1.80)	1.30 (0.90-2.00)	1.40 (0.90-2.40)	1.50 (1.00-2.60)	<0.001
Hematocrit(%)	31.20 (26.80-35.80)	29.80 (25.80-34.30)	31.20 (26.92-35.68)	31.90 (27.50-36.50)	32.10 (27.20-36.70)	<0.001
Hemoglobin(g/dL)	10.10 (8.60-11.70)	9.60 (8.30-11.20)	10.10 (8.70-11.70)	10.30 (8.80-11.85)	10.40 (8.80-11.90)	<0.001
Lactate(mmol/L)	2.00 (1.40-3.10)	1.70 (1.20-2.60)	1.80 (1.30-2.70)	2.10 (1.40-3.20)	2.40 (1.60-4.20)	<0.001
ALT(IU/L)	30.00 (17.00-65.00)	20.00 (12.00-39.00)	26.00 (16.00-46.00)	31.00 (18.00-61.00)	56.00 (27.00-163.25)	<0.001
AST(IU/L)	143.00 (24.00-96.00)	25.00 (17.00-45.00)	34.00 (23.00-62.50)	47.00 (29.00-92.00)	104.00 (47.00-288.25)	<0.001
PLT(*10^9^/L)	180.00 (115.00-264.00)	191.00 (122.50-281.00)	185.00 (121.00-267.00)	178.50 (114.25-258.00)	169.00 (99.00-250.00)	<0.001
PT(s)	15.30 (13.30-19.90)	15.00 (13.20-18.20)	15.00 (13.10-19.20)	15.70 (13.50-20.60)	15.80 (13.50-21.60)	<0.001
TT(s)	332.90 (28.70-40.50)	32.40 (28.90-38.77)	32.40 (28.55-38.90)	33.00 (28.50-41.00)	34.10 (29.00-44.30)	<0.001
RBC(*10^12^/L)	3.40 (2.90-3.94)	3.28 (2.81-3.79)	3.37 (2.91-3.92)	3.47 (2.96-3.99)	3.47 (2.91-4.07)	<0.001
Sodium(mmol/L)	138.00 (134.00-141.00)	137.00 (134.00-140.00)	138.00 (134.00-141.00)	138.00 (135.00-141.00)	138.00 (134.00-141.00)	0.030
Urea nitrogen(mg/dL)	28.00 (18.00-47.00)	24.00 (15.00-40.00)	27.00 (17.00-45.00)	30.00 (19.00-49.00)	32.00 (21.00-53.00)	<0.001
WBC(*10^9^/L)	12.20 (7.60-18.00)	10.90 (6.60-16.30)	11.90 (7.60-17.00)	12.90 (8.10-19.10)	12.90 (8.30-19.60)	<0.001
**Scoring systems(IQR)**						
SOFA	3.00 (2.00-4.00)	2.00 (2.00-4.00)	2.00 (2.00-4.00)	3.00 (2.00-4.00)	3.00 (2.00-5.00)	<0.001
APACHEII	12.00 (9.00-15.00)	11.00 (8.00-14.00)	11.00 (9.00-14.00)	12.00 (9.00-15.00)	12.00 (10.00-16.00)	<0.001
**Clinical outcomes(days)**						
LOS in ICU	4.78 (2.16-10.85)	3.63 (1.90-8.44)	4.32 (2.10-10.24)	5.15 (2.42-11.09)	6.09 (2.71-13.29)	<0.001
LOS in hospital	11.15 (6.21-20.71)	10.30 (5.89-20.11)	10.94 (6.59-19.74)	11.82 (6.75-21.64)	11.91 (5.92-21.24)	0.017
**30-day mortality(n,%)**	1870 (27.60%)	256 (15.19%)	355 (20.92%)	494 (29.08%)	765 (45.16%)	<0.001
**1-year mortality(n,%)**	2384 (35.19%)	385 (22.85%)	480 (28.29%)	626 (36.85%)	893 (52.72%)	<0.001

**Abbreviations:** LDH = lactate dehydrogenase, CAD = coronary artery disease, SBP = systolic blood pressure, DBP = diastolic blood pressure, HR = heart rate, RR = respiratory rate, WBC = white blood cells, PLT = platelet, RBC = red blood cells, PT = prothrombin time, TT = thrombin time, AG = anion gap, ALT = alanine aminotransferase, AST = aspartate aminotransferase, SOFA = sequential organ failure assessment, APACHE = acute physiology and chronic health evaluation, LOS = length of stay, ICU = intensive care unit, IQR = interquartile ranges.

Univariable analysis was utilized for exploring the associations of different variables with 30-day mortality and 1-year mortality. Three models were constructed for explore the association of LDH with clinical outcomes: crude model (adjusted for none), model I (adjusted for age and gender) and model II (adjusted for all potential confounders). Moreover, LDH was changed to be a categorical variable (quartiles), and the P value for trend of categorized LDH was calculated. Then, a generalized additive model and a smooth fitting curve were performed. If nonlinearity was found, the inflection point of LDH was confirmed by recursive algorithm. On the basis of the P value of the log-likelihood ratio test, the better fitting model was confirmed. If the P value <0.05, the nonlinear model was selected. The receiver-operator characteristic (ROC) analysis of LDH for predicting mortalities of 30-day and 1-year were performed. Predictive performances including specificity, sensitivity and cut-off value were calculated.

The statistical software of EmpowerStats (http://www.empowerstats.com) and R (http://www.R-project.org) were applied for analysis. A P-value less than 0.05 was confirmed as statistically significant.

## Results

### General characteristics of the patients

Based on exclusion criteria, 6775 sepsis patients were finally included (S1 Fig). Basic characteristics of the cohort were demonstrated in Table 1. The median age was 66 and males accounted for 55.47% (n = 3758). Mortalities of 30-day and 1-year were 27.60%(n = 1870) and 35.19%(n = 2384). The median scores of SOFA and APAHCEII were 3 and 12, respectively.

Table 1 also clarified the different variables between Q1-Q4 groups based on LDH quartiles. Significant differences were found in the variables except gender(P = 0.254), CAD (P = 0.207), diabetes(P = 0.499), and hypertension(P = 0.182). In Q4 group, mortalities of 30-day and 1-year were 45.16% and 52.72%, respectively.

### Univariate analysis for 30-day mortality and 1-year mortality

In Table 2, variables including age, renal disease, RR, RBC, total bilirubin, LDH, AG, SBP, DBP, lactate, creatinine, PT, TT, hemoglobin, urea nitrogen, ALT, AST, PLT, sodium, APACHEII and SOFA were both related with 30-day and 1-year mortality by univariate analysis.

**Table 2:** Univariate analysis for 30-day mortality and 1-year mortality.

Variables	Univariate (OR,95%CI, P-value) (30-day mortality)	Univariate (OR,95%CI, P-value) (1-year mortality)
Age(years)	1.02 (1.01, 1.02) <0.0001	1.01 (1.01, 1.02) <0.0001
Gender		
Male	Ref.	Ref.
Female	1.01 (0.91, 1.13) 0.8157	0.97 (0.88, 1.08) 0.5864
Renal disease		
No	Ref.	Ref.
Yes	1.33 (1.04, 1.70) 0.0228	1.66 (1.32, 2.10) <0.0001
CAD		
No	Ref.	Ref.
Yes	1.15 (0.96, 1.38) 0.1412	1.22 (1.03, 1.45) 0.0207
Diabetes		
No	Ref.	Ref.
Yes	0.76 (0.54, 1.07) 0.1212	0.89 (0.65, 1.21) 0.4544
Hypertension		
No	Ref.	Ref.
Yes	0.96 (0.84, 1.10) 0.5363	0.89 (0.78, 1.00) 0.0569
HR(beats/min)	1.00 (1.00, 1.00) 0.1951	1.00 (1.00, 1.00) 0.0948
SBP(mmHg)	1.00 (0.99, 1.00) 0.0057	1.00 (0.99, 1.00) 0.0020
DBP(mmHg)	1.00 (0.99, 1.00) 0.0160	1.00 (0.99, 1.00) 0.0025
RR(beats/min)	1.02 (1.01, 1.02) <0.0001	1.01 (1.01, 1.02) 0.0011
LDH(per100IU/L increment)	1.11 (1.10, 1.13) <0.0001	1.11 (1.10, 1.13) <0.0001
AG(mmol/l)	1.06 (1.05, 1.07) <0.0001	1.05 (1.04, 1.06) <0.0001
Total bilirubin(mg/dL)	1.06 (1.05, 1.07) <0.0001	1.06 (1.04, 1.07) <0.0001
Total calcium(mg/dL)	1.02 (0.96, 1.08) 0.5761	1.07 (1.01, 1.13) 0.0164
Creatinine(mg/dL)	1.08 (1.05, 1.11) <0.0001	1.08 (1.05, 1.11) <0.0001
Hematocrit(%)	1.00 (0.99, 1.01) 0.5235	0.99 (0.98, 1.00) 0.0020
Hemoglobin(g/dL)	0.98 (0.95, 1.00) 0.0478	0.95 (0.93, 0.97) <0.0001
Lactate(mmol/L)	1.23 (1.20, 1.26) <0.0001	1.20 (1.17, 1.23) <0.0001
ALT(IU/L)	1.00 (1.00, 1.00) <0.0001	1.00 (1.00, 1.00) 0.0027
AST(IU/L)	1.00 (1.00, 1.00) <0.0001	1.00 (1.00, 1.00) <0.0001
PLT(*10^9^/L)	1.00 (1.00, 1.00) <0.0001	1.00 (1.00, 1.00) <0.0001
PT(s)	1.02 (1.01, 1.02) <0.0001	1.02 (1.01, 1.02) <0.0001
TT(s)	1.01 (1.01, 1.01) <0.0001	1.01 (1.01, 1.01) <0.0001
RBC(*10^12^/L)	0.85 (0.79, 0.92) <0.0001	0.80 (0.75, 0.86) <0.0001
Sodium(mmol/L)	0.99 (0.98, 1.00) 0.0024	0.98 (0.98, 0.99) 0.0002
Urea nitrogen(mg/dL)	1.01 (1.01, 1.01) <0.0001	1.01 (1.01, 1.01) <0.0001
WBC(*10^9^/L)	1.01 (1.00, 1.01) 0.0386	1.00 (0.99, 1.00) 0.8444
APAHCEII	1.11 (1.09, 1.12) <0.0001	1.10 (1.08, 1.11) <0.0001
SOFA	1.21 (1.18, 1.24) <0.0001	1.20 (1.18, 1.23) <0.0001

**Abbreviations:** LDH = lactate dehydrogenase, CAD = coronary artery disease, SBP = systolic blood pressure, DBP = diastolic blood pressure, HR = heart rate, RR = respiratory rate, WBC = white blood cells, PLT = platelet, RBC = red blood cells, PT = prothrombin time, TT = thrombin time, AG = anion gap, ALT = alanine aminotransferase, AST = aspartate aminotransferase, SOFA = sequential organ failure assessment, APACHE = acute physiology and chronic health evaluation, OR=odds ratio, CI = confidential interval.

### Relationship between LDH and mortalities of 30-day and 1-year

In Table 3, three models including unadjusted model (crude model) and adjusted models (model I and model II) were performed for investigating the association of LDH with prognosis. In model II (adjusted for all potential confounders), for every 100 IU/L increment in LDH, the risk of 30-day mortality and 1-year mortality increased by 11% (odds ratio (OR)=1.11, 95%CI:1.08–1.13, P < 0.0001) and 12% (OR=1.12, 95%CI: 1.09–1.14, P < 0.0001), respectively. In addition, we also analyzed the relationship between LDH (categorial variables(Q1-Q4)) and clinical outcomes. In Q4 group of Model II, the risk of 30-day mortality and 1-year mortality increased the most and the values of OR were 3.51 (95%CI:2.87–4.29, P < 0.0001) and 3.19 (95%CI:2.65–3.83, P < 0.0001), respectively.

**Table 3:** Relationship between LDH and clinical outcomes in different models.

Exposure	Crude model(OR, 95%CI, P)	Model I(OR, 95%CI, P)	Model II (OR, 95%CI, P)
**30-day mortality**			
LDH(per 100 IU/L increment)	1.11 (1.10, 1.13) <0.0001	1.12 (1.10, 1.13) <0.0001	1.11 (1.08, 1.13) <0.0001
LDH(IU/L) quartiles			
Q1	Ref.	Ref.	Ref.
Q2	1.48 (1.24, 1.76) <0.0001	1.41 (1.18, 1.68) 0.0002	1.44 (1.17, 1.76) 0.0006
Q3	2.29 (1.93, 2.71) <0.0001	2.22 (1.87, 2.63) <0.0001	1.86 (1.52, 2.28) <0.0001
Q4	4.60 (3.90, 5.42) <0.0001	4.69 (3.98, 5.54) <0.0001	3.51 (2.87, 4.29) <0.0001
P for trend	<0.0001	<0.0001	<0.0001
**1-year mortality**			
LDH(per 100 IU/L increment)	1.11 (1.10, 1.13) <0.0001	1.12 (1.10, 1.13) <0.0001	1.12 (1.09, 1.14) <0.0001
LDH(IU/L) quartiles			
Q1	Ref.	Ref.	Ref.
Q2	1.33 (1.14, 1.56) 0.0003	1.29 (1.10, 1.51) 0.0015	1.34 (1.12, 1.61) 0.0016
Q3	1.97 (1.69, 2.29) <0.0001	1.93 (1.66, 2.24) <0.0001	1.73 (1.44, 2.07) <0.0001
Q4	3.76 (3.25, 4.37) <0.0001	3.81 (3.28, 4.42) <0.0001	3.19 (2.65, 3.83) <0.0001
P for trend	<0.0001	<0.0001	<0.0001

Crude model adjusted for: None;

Model I adjusted for: age; gender;

Model II adjusted for: age; gender; HR; SBP; DBP; RR; AG; ALT, AST, total bilirubin; total calcium; creatinine; hematocrit; hemoglobin; PLT; PT; TT; RBC; urea nitrogen; lactate; WBC; sodium; renal disease; CAD; diabetes; hypertension; SOFA; APAHCEII.

**Abbreviations:** LDH = lactate dehydrogenase, CAD = coronary artery disease, SBP = systolic blood pressure, DBP = diastolic blood pressure, HR = heart rate, RR = respiratory rate, WBC = white blood cells, PLT = platelet, RBC = red blood cells, PT = prothrombin time, TT = thrombin time, AG = anion gap, ALT = alanine aminotransferase, AST = aspartate aminotransferase, SOFA = sequential organ failure assessment, APACHE = acute physiology and chronic health evaluation, OR=odds ratio, CI = confidential interval.

### A nonlinear association between LDH and clinical outcomes in sepsis

**Fig 1:**
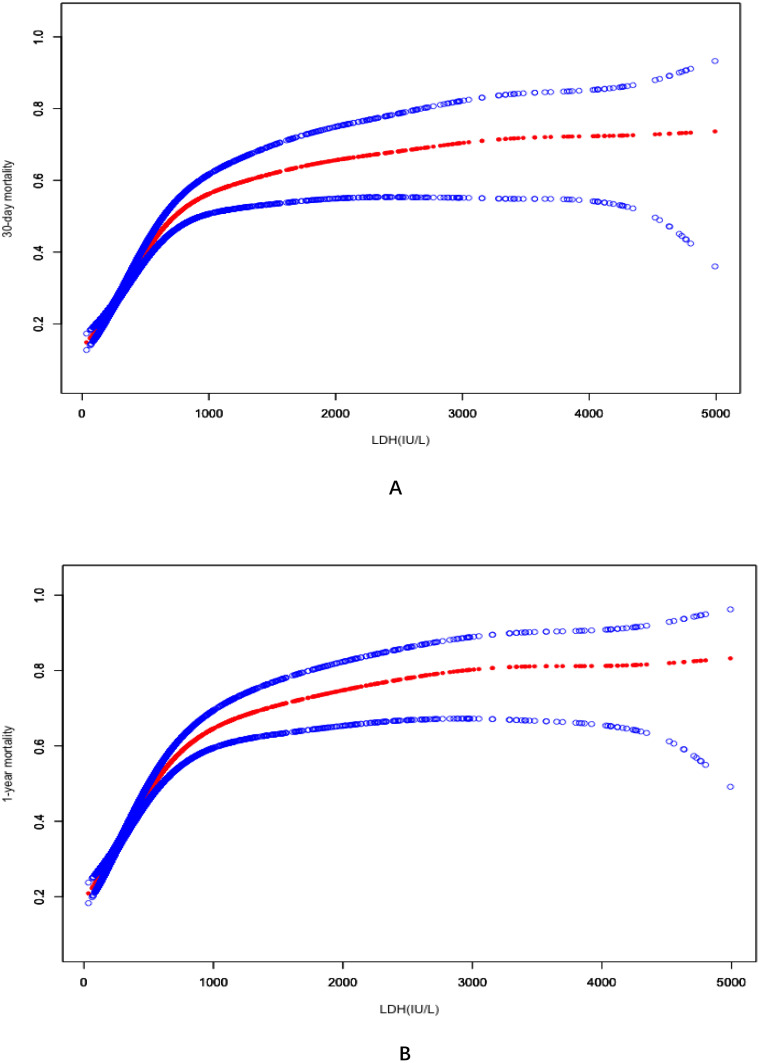
Smooth fitting curves identified the non-linear relationships between LDH and mortalities of 30-day (A) and 1-year (B) in sepsis. Abbreviation: LDH = lactate dehydrogenase.

Two models including the linear model and non-linear model were applied for fitting the association (S1 Table). The nonlinear model was confirmed based on it’s higher accuracy (both P < 0.001). Smooth fitting curves showed that the nonlinear associations between LDH and clinical outcomes (30-day mortality in Fig 1A and 1-year mortality in Fig 1B) in sepsis were identified after adjusted for all the potential confounders.

The inflection point of LDH in 30-day mortality and 1-year mortality were 625 IU/L and 638 IU/L. For 30-day mortality, the values of OR in slope 1 (left side) and slope 2 (right side) were 1.35(95%CI:1.29–1.42, P < 0.0001) and 1.03 (95%CI: 1.01–1.05, P = 0.0023), respectively. For 1-year mortality, the values of OR in slope 1 (left side) and slope 2 (right side) were 1.33(95%CI:1.27–1.39, P < 0.0001) and 1.04 (95%CI: 1.02–1.06, P = 0.0006), respectively.

### Predictive performances of LDH for clinical outcomes

**Fig 2:**
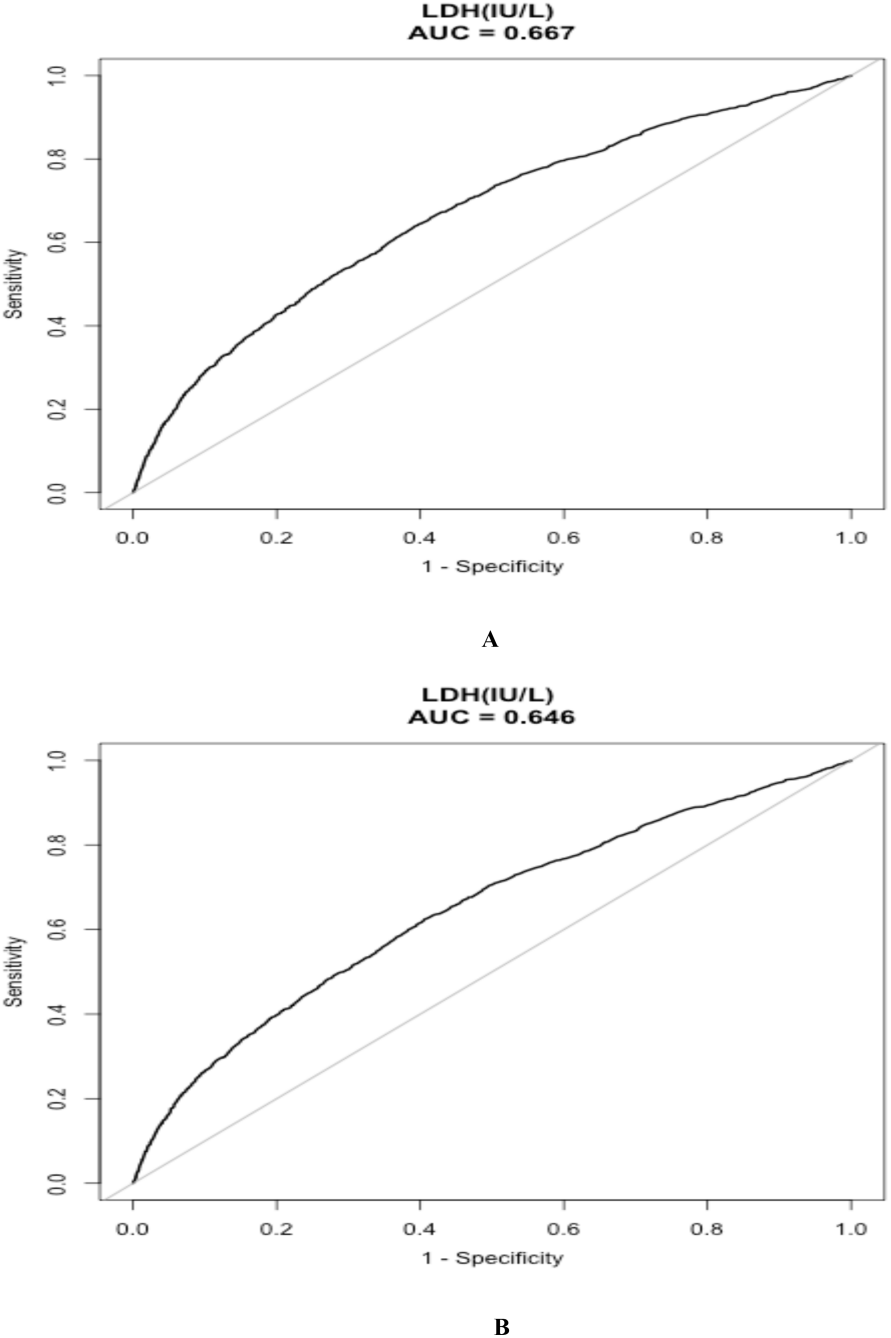
ROCs of LDH for predicting 30-day mortality(A) and 1-year mortality(B) in sepsis. Abbreviation: LDH = lactate dehydrogenase.

In Table 4, predictive performances of LDH in clinical outcomes were compared. The areas under the ROC curve (AUC) of LDH for predicting mortalities of 30-day and 1-year were 0.667 (95%CI:0.652–0.681), and 0.646 (95%CI: 0.632–0.660), respectively. The cut-off values of LDH for 30-day mortality and 1-year mortality were 280 IU/L and 272 IU/L, respectively. ROCs of LDH for predicting mortalities of 30-day and 1-year in sepsis were demonstrated in Fig 2.

**Table 4:** Predictive performances of LDH for clinical outcomes.

Variables	AUC	95%CI lower	95%CI upper	Cut-off value	Specificity	Sensitivity
**30-day mortality**						
LDH(IU/L)	0.667	0.652	0.681	280	0.608	0.638
**1-year mortality**						
LDH(IU/L)	0.646	0.632	0.660	272	0.593	0.624

**Abbreviations:** AUC = area under the curve, CI = confidential interval, LDH = lactate dehydrogenase.

## Discussion

In our study, the nonlinear relationship between LDH and mortalities of 30-day and 1-year in sepsis was found and LDH was positively associated with poor prognosis. For every 100 IU/L increment in LDH, the risk of 30-day mortality and 1-year mortality increased by 11% and 12%, respectively. Moreover, LDH had a predictive value for clinical outcomes.

In recent years, a cumulating large number of data which have been created from the comprehensive utility of electronic medical records have been utilized for helping clinicians in making individualized decision and improving outcomes for patients [26–28]. At present, the diagnosis and evaluation of sepsis are mainly based on the assessment of organ dysfunction, including qSOFA and SOFA score [29]. Other scoring systems include APAHCEII, NEWS, etc., which have certain applications in sepsis [30,31]. The commonly used laboratory examination indicators currently include blood routine, liver and kidney function, and inflammation indicators, all of which have a certain correlation with the prognosis of sepsis [32–34].Although the case fatality rate of sepsis is comparatively high, early identification of those patients with higher risk of poor prognosis is still a big challenge, and the generalized applicability of prognostic factors is limited [35].

In sepsis, few researches have been done for exploring the prognostic value of LDH. Usually, LDH was analyzed as one of elements in predictive models. One Australian study investigated more than one hundred variables in sepsis and constructed a 4-Hour Cairns Sepsis Model, while LDH was one of the ten variables included in the model [36]. Another deep-learning research for identifying sub-phenotypes in sepsis illuminated that LDH was a basic parameter for performing the differentiating models [37]. One recent research including 192 patients in China found that LDH was an independent risk factor for 28-day death of sepsis (HR = 1.005, 95% CI: 1.002–1.007, P = 0.001) [38]. Another research from University of Oklahoma showed that increased LDH was associated with higher risk of physiologic abnormalities and organ failure in sepsis [39]. LDH also could be a biomarker for differentiating the sepsis, severe sepsis and septic shock based on the old and new criteria [40]. Although some previous studies also analyzed the relationship between LDH and prognosis in sepsis, these studies only included comparatively small number samples of sepsis patients. Our research comprehensively analyzed the clinical and laboratory factors in more than several thousands sepsis patients and found the positive relationship between LDH and mortalities of short-term and long-term.

Several plausible explanations could be elucidated the relationship between LDH and sepsis. First, severe inflammatory responses are activated in sepsis and a great amount of inflammatory markers are produced [41]. LDH has been proved to be a potential biomarker for inflammation, which is associated with the endothelium dysfunction and imbalanced microcirculation perfusion, leading to the pathological progression of worse prognosis [42]. Second, hypoxia usually occurs during sepsis. Oxygen deficiency can activate the expression of LDH and results in production of lactate, while the latter is linked with outcomes [43]. Third, the increased amounts of the injured cells including apoptosis and necrosis due to infection cause the leakage of LDH into the blood, which could be detected as a significant elevated level [44].

Some limitations in our study should be declared. First, due to some missing data in the database, factors including the sites of infection, socioeconomic status and some inflammatory markers such as C-reactive protein couldn’t be evaluated. Second, although we have tried our best to adjust for confounding factors, there may still be some prognostic confounding factors that have not been adjusted. Meanwhile, there may be selection bias in studies related to age and comorbidities in the 1-year mortality risk. Second, the conclusions, which were made based on MIMIC-IV, haven’t been externally validated. Further prospective research with multiple-centers should be performed for validation. Third, the database didn’t explicit the missing information of post-discharge outcomes, so it might be the limitation of our study. Fourth, our study couldn’t prove a causal relationship between LDH and the prognosis of sepsis, but only indicates a correlation. The specific mechanism still needs further research.

## Conclusion

LDH was positively associated with 30-day mortality and 1-year mortality in sepsis and the relationship was nonlinear. LDH has a prognostic value for clinical outcomes in sepsis. Our results could enable clinicians to screening out the patients with worse outcomes so that individualized management including medical treatment and nursing care could be done timely for those patients.

## Supporting information

S1 Fig Flow chart for study design. Abbreviation: LDH = lactate dehydrogenase.
(PDF)

S1 Table Comparison of linear and non-linear models between LDH and clinical outcomes. (DOCX)
