# Supplementary figures and images for: Lactate dehydrogenase is an indicator for outcomes of short-term and long-term in septic patients
Source: PLoS One. 2025 Dec 8;20(12):e0337213. doi: 10.1371/journal.pone.0337213 (PMC12685224; doi:10.1371/journal.pone.0337213)

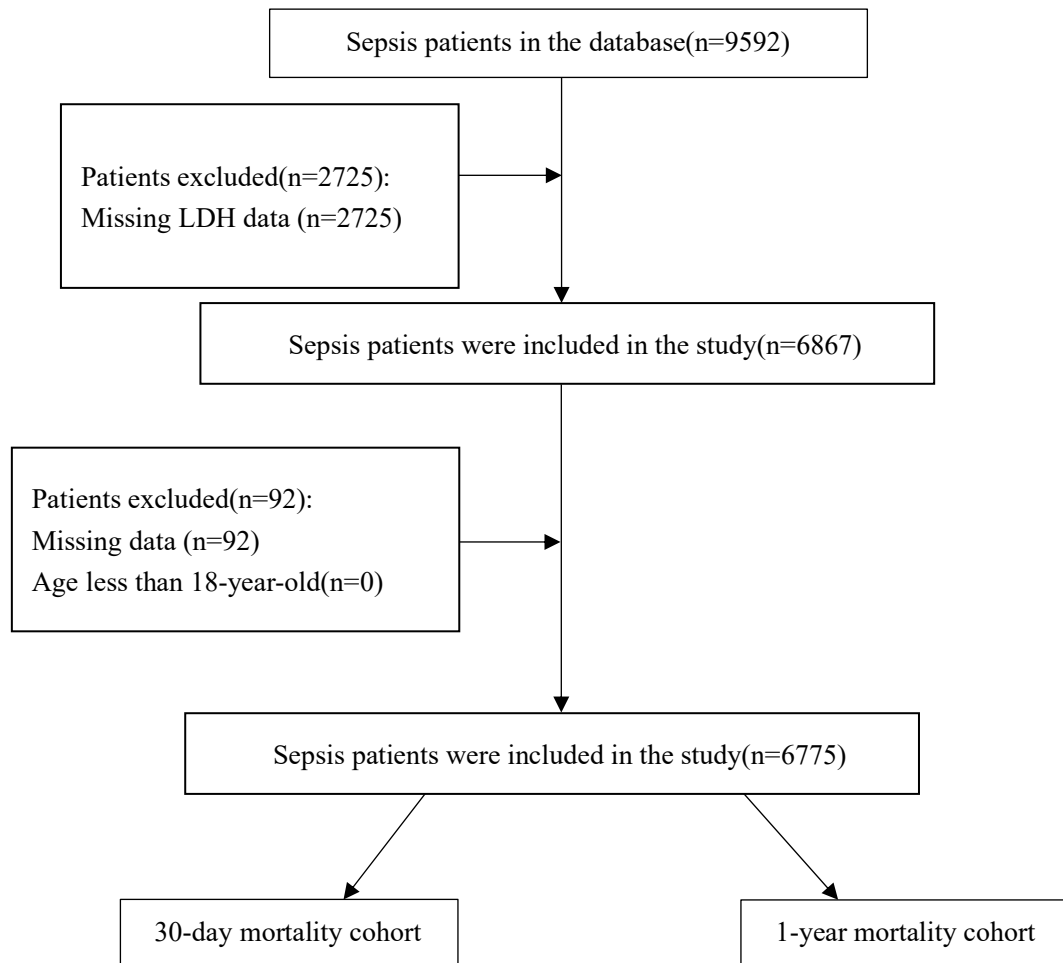

Supplementary Figure 1: Flow chart for study design.

Abbreviation: LDH= lactate dehydrogenase.

Supplement: S1 Fig — Abbreviation: LDH = lactate dehydrogenase. (PDF) [file pone.0337213.s001.pdf]
