# Supplementary material for: Lactate dehydrogenase is an indicator for outcomes of short-term and long-term in septic patients
Source: PLoS One. 2025 Dec 8;20(12):e0337213. doi: 10.1371/journal.pone.0337213 (PMC12685224; doi:10.1371/journal.pone.0337213)
Supplement: S1 Table — (DOCX) [file pone.0337213.s002.docx]

**Supplementary Table 1 Comparison of linear and non-linear models between LDH and clinical outcomes**

|  | Number(%) | OR(95%CI), P-value |
| --- | --- | --- |
| **30-day mortality** |  |  |
| Model I: The linear model | 6775(100%) | 1.11 (1.08, 1.13) <0.0001 |
| Model II: Two-segment non-linear model |  |  |
| The turning point of LDH (IU/L) |  |  |
| ≤ 625(slope 1: left side) | 6060(89.45%) | 1.35 (1.29, 1.42) <0.0001 |
| > 625(slope 2: right side) | 715(10.55%) | 1.03 (1.01, 1.05) 0.0023 |
| Slope 2 to slope 1 |  | 0.76 (0.72, 0.81) <0.0001 |
| Predicted at 625 |  | 0.06 (-0.08, 0.20) |
| P for the log-likelihood ratio test |  | <0.001 |
| **1-year mortality** |  |  |
| Model I: The linear model | 6775(100%) | 1.12 (1.09, 1.14) <0.0001 |
| Model II: Two-segment non-linear model |  |  |
| The turning point of LDH(IU/L) |  |  |
| ≤ 638(slope 1:left side) | 6089(89.87%) | 1.33 (1.27, 1.39) <0.0001 |
| > 638(slope 2:right side) | 686(10.13%) | 1.04 (1.02, 1.06) 0.0006 |
| Slope 2 to slope 1 |  | 0.78 (0.74, 0.83) <0.0001 |
| Predicted at 638 |  | 0.38 (0.24, 0.52) |
| P for the log-likelihood ratio test |  | <0.001 |

Model adjusted for: age; gender; HR; SBP; DBP; RR; AG; ALT, AST, total bilirubin; total calcium; creatinine; hematocrit; hemoglobin; PLT; PT; TT; RBC; urea nitrogen; lactate; WBC; sodium; renal disease; CAD; diabetes; hypertension; SOFA; APAHCEII.

**Abbreviations:** LDH=lactate dehydrogenase, CAD=coronary artery disease, SBP=systolic blood pressure, DBP= diastolic blood pressure, HR= heart rate, RR=respiratory rate, WBC=white blood cells, PLT=platelet, RBC=red blood cells, PT=prothrombin time, TT=thrombin time, AG=anion gap, ALT=alanine aminotransferase, AST= aspartate aminotransferase, SOFA=sequential organ failure assessment, APACHE=acute physiology and chronic health evaluation, OR=odds ratio, CI= confidential interval.
